# The reproductive system of *Osedax* (Annelida, Siboglinidae): ovary structure, sperm ultrastructure, and fertilization mode

**DOI:** 10.1111/ivb.12037

**Published:** 2013-12-06

**Authors:** Sigrid Katz, Greg W Rouse

**Affiliations:** 1 Scripps Institution of Oceanography, University of California San DiegoLa Jolla, California, 92093-0202, USA

**Keywords:** reproduction, sperm storage, morphology, heart body

## Abstract

*Osedax* is a genus of siboglinid annelids in which the females live on dead vertebrate bones on the seafloor. These females have a posterior end that lies within the bone and contains the ovarian tissue, as well as the “roots” involved with bone degradation and nutrition. The males are microscopic and live as “harems” in the lumen of the gelatinous tube that surrounds the female trunk, well away from the ovary. Females are known to spawn fertilized primary oocytes, suggesting internal fertilization. However, little is known about sperm transfer, sperm storage, or the location of fertilization, and the morphology of the female reproductive system has not been described and compared with the reproductive systems of other siboglinids. A 3D-reconstruction of the ovisac of *Osedax* showed ovarian tissue with multiple lobes and mature oocytes stored in a “uterus” before being released through the single oviduct. The oviduct emerges as a gonopore on the trunk and travels along the trunk to finally open to the seawater as a thin cylindrical tube among the crown of palps. Light and transmission electron microscopy of mature *Osedax* sperm revealed elongate heads consisting of a nucleus with helical grooves occupied by mitochondria. In contrast to other Siboglinidae, *Osedax* sperm are not packaged into spermatophores or spermatozeugmata, and *Osedax* females lack a discrete region for sperm storage. Transmission electron microscopy and fluorescence microscopy allowed detection of sperm associated with ovarian tissue of the female ovisac of four different *Osedax* species. This provides the first evidence for the site of internal fertilization in *Osedax*. A heart body was found in the circulatory system, as seen in other siboglinids and some other annelids. The possible presence of nephridia in the anterior ovisac region was also documented. These morphological features provide new insights for comparing the regionalization of *Osedax* females in relation to other siboglinids.

Siboglinids are found at unusual marine habitats such as cold seeps, hydrothermal vents, wood falls, and whale falls (Rouse et al. 2004; Southward et al. 2005; Bright & Lallier 2010). The group has become well known for being essentially gutless worms living in symbiosis with thiotrophic, methanotrophic, or heterotrophic bacteria to provide nutrition (Rouse et al. 2004; Goffredi et al. 2005; Bright & Lallier 2010). Siboglinidae currently comprise the taxa Monilifera (containing Vestimentifera and *Sclerolinum*), Frenulata (containing most of what was formerly regarded as Pogonophora), and *Osedax* Rouse,Goffredi & Vrijenhoek 2004 (Rouse 2001; Rouse et al. 2004; Hilário et al. 2011). Although some siboglinid taxa have been known for decades, detailed information on their reproductive biology is limited because they live in habitats that are difficult to access.

The most recently discovered group of Siboglinidae is *Osedax*, which occurs on bones lying on the seafloor (Rouse et al. 2004; Glover et al. 2005; Fujikura et al. 2006; Amon et al. 2013). All *Osedax* species exhibit conspicuous sexual dimorphism with larger females and dwarf males (Rouse et al. 2008, 2009). Females have an anterior retractable trunk, usually ending with a crown of four palps and an oviduct. The trunk is enclosed in a gelatinous tube, into which the crown may also be retracted. The region immediately following the trunk is called the ovisac and is located beneath the bone surface. The ovisac houses the ovarian tissue and is ensheathed by an epidermis, a muscle layer, and trophosomal tissue, the latter containing endosymbiotic Oceanospirillales bacteria. The tissue layers ensheathing the ovisac also ramify further into the bone, producing “root” structures (Rouse et al. 2004; Goffredi et al. 2005; Katz et al. 2011). *Osedax* males are microscopic dwarfs resembling larval forms and contain spermatids, sperm, and yolk droplets. Males may accumulate in high numbers in the lumen of the female tubes (Rouse et al. 2004, 2009; Vrijenhoek et al. 2008; Worsaae & Rouse 2010).

There is some variation in the organization of the female reproductive systems among the different siboglinid taxa (Table 1). Most siboglinids, including *Osedax*, are gonochoric and the sexes can be differentiated by the location of the gonopores and the presence or absence of external genital grooves (Ivanov 1963; Gardiner & Jones 1993; Southward 1993; Southward et al. 2005; Eichinger et al. 2013), although only *Osedax* exhibits dramatic sexual dimorphism. From the gonopores at the anterior end of the worms, the oviducts run posteriorly inside the trunk tissue and parallel to the ovaries, which can span part or the whole length of the trunk (Webb 1977; Hilário et al. 2005; Karaseva et al. 2012), resulting in a U-shaped reproductive system. A germinal epithelium producing oocytes can be found along each ovary and these oocytes are retained in the ovary until they are mature. The mature oocytes enter the oviduct at the posterior end of the ovary. Just before the gonopores, the oviducts expand (referred to as ovisacs in Vestimentifera) and hold fertilized eggs before spawning (Bakke 1976; Webb 1977; Gardiner & Jones 1993; Southward 1999; Hilário et al. 2005; Karaseva et al. 2012).

**Table 1:** Aspects of reproductive systems in Siboglinidae.

	Frenulata	*Osedax*	Monilifera (*Sclerolinum*)	Monilifera (Vestimentifera)
Female gonad shape	U-shaped (Ivanov 1961)	Somewhat linear, but coiled and lobed (this study)	U-shaped (Eichinger et al. 2013)	U-shaped (Webb 1977; Malakhov et al. 1996; Hilário et al. 2005; Karaseva et al. 2012)
Number of ovaries	One pair (Ivanov 1961)	?; multiple ovarian lobes present (this study)	Single ovary (Eichinger et al. 2013)	One pair, one may be larger (Webb 1977; Gardiner & Jones 1993; Hilário et al. 2005; Karaseva et al. 2012)
Location of ovaries	Along trunk region (Ivanov 1961)	In ovisac region (Rouse et al. 2004; this study)	Along trunk region (Eichinger et al. 2013)	Along trunk region (Webb 1977; Gardiner & Jones 1993; Hilário et al. 2005; Karaseva et al. 2012)
Oviduct/s	One pair (Ivanov 1961)	Single oviduct (Rouse et al. 2004; this study)	Single oviduct (Eichinger et al. 2013)	One pair (Webb 1977; Malakhov et al. 1996; Hilário et al. 2005; Karaseva et al. 2012)
Storage of mature/fertilized eggs	Eggs and embryos in distal part of oviducts, just before gonopores (Bakke 1976; Southward 1999)	Uterus region of the oviduct (this study)	Lateral pouch at distal part of ovary (Eichinger et al. 2013)	Ovisac region of oviducts, just before gonopores (Webb 1977; Gardiner & Jones 1993; Hilário et al. 2005; Karaseva et al. 2012)
Female gonopores	Paired, dorsally on preannular region of the trunk (Ivanov 1961; Southward et al. 2005)	One, dorsally on trunk; and continues forward to exit in crown (Rouse et al. 2004; this study)	One, dorsally on anterior trunk	Paired, dorsally on anterior trunk (Gardiner & Jones 1993; Southward et al. 2005)
Sperm storage by females	No direct evidence	In ovarian lobes (this study)	No evidence	Spermathecal region of oviduct, just outside ovary (Gardiner & Jones 1985; Malakhov et al. 1996; Hilário et al. 2005; Drozdov & Galkin 2012; Karaseva et al. 2012)
Sperm packaging by males	Spermatophores (Ivanov 1963; Flügel 1977)	None, free sperm (Rouse et al. 2004; this study)	Spermatozeugmata or free (Southward et al. 2005)	Spermatozeugmata (Southward & Coates 1989; Marotta et al. 2005)

Siboglinid sperm are filiform, with an elongate head and flagellum. They possess a helically grooved nucleus with elongate mitochondria occupying this groove (Franzén 1973; Jones & Gardiner 1985; Rouse et al. 2004). Despite this similarity, the sperm are packaged in different ways (Table 1). Frenulate sperm are encapsulated as spermatophores, with long filaments at one end (Ivanov 1963; Flügel 1977), while vestimentiferan sperm are bundled into spermatozeugmata (Southward & Coates 1989), a feature found in several other annelid groups (Rouse 1999). Both frenulate spermatophores and vestimentiferan spermatozeugmata can be sticky (Southward & Coates 1989; Marotta et al. 2005), allowing attachment to surfaces such as female tubes (Bakke 1991). Direct transfer of spermatophores or spermatozeugmata from male to female may occur (Webb 1963; Southward & Coates 1989; MacDonald et al. 2002; Southward et al. 2005), but release of gametes into the water column by males and females also has been observed for the vestimentiferan *Riftia pachyptila* Jones 1981 (Van Dover 1994; Hilário et al. 2005). Little is known about the reproductive biology of *Sclerolinum*, although the sperm do not appear to be packaged in spermatophores (Southward et al. 2005).

Early evidence for internal fertilization in siboglinids was Bakke’s (1976) observation that eggs removed from the oviducts of the frenulate *Siboglinum fiordicum* Webb 1963 would begin cleavage without the addition of sperm. Subsequently, spermatozoa were found stored along the posterior oviduct of vestimentiferan females, near the entrance to the ovary (Jones & Gardiner 1985; Hilário et al. 2005; Karaseva et al. 2012). *Osedax* females contain fertilized primary oocytes that are arrested in development until after spawning, which provides indirect evidence for internal fertilization (Rouse et al. 2008, 2009).

The morphology of the *Osedax* female reproductive system has yet to be described. It is known that sperm accumulate in the head of males (Worsaae & Rouse 2010), but how sperm are transferred to the female and where fertilization occurs are unknown. Here, we describe the morphology of the female reproductive system and the mature sperm of males, compare these features with those of other siboglinids, assess the evidence for internal fertilization in *Osedax*, and investigate the method of fertilization.

## Methods

### Samples

Specimens were collected from bones in the Monterey Canyon off the coast of California using the remotely operated vehicles (ROVs) *Tiburon* or *Doc Ricketts* (on the ship R/V *Western Flyer*) during August and December 2007, March 2009, and October 2010. *Osedax rubiplumus* Rouse,Goffredi & Vrijenhoek 2004 was collected from bones of Whale-1820 (depth 1820 m) during dive T1119. An undescribed species referred to as *Osedax* “green palp” was collected from cow bones from the same site during dive T1163. *Osedax frankpressi* Rouse,Goffredi & Vrijenhoek 2004 was collected from Whale-2892 during dive DR10 and DR204. Another undescribed species referred to as *Osedax* “yellow collar” was collected from seal bones at around 633 m depth during dive DR205 and from whale bones from Whale-600 during dive DR207.

### Transmission electron microscopy

*Osedax rubiplumus* and *O*. “green palp” (T1163 and T1119) females were fixed as a whole in a mixture of 1.5% acrolein, 3% glutaraldehyde, and 1.5% paraformaldehyde in 0.1M cacodylate buffer (ph 7.4) containing 10% (w/v) sucrose overnight. Males of *O. rubiplumus* were fixed in place in female tubes in 3% glutaraldehyde in 0.2 mol L^−1^ cacodylate buffer (pH 7.4) with 0.3 mol L^−1^ sucrose for 2 h. Specimens were rinsed in the same buffer as used for fixation three times for 10 min, post-fixed in 1 or 2% osmium tetroxide (OsO_4_) in buffer for 1–2 h, rinsed again with the same buffer three times for 10 min, and dehydrated with a graded ethanol series up to 70%. All steps were carried out on ice. Specimens were stored in 70% ethanol at 4–8°C until embedding. Specimens were dehydrated in an ethanol series and embedded in either Spurr’s or AGAR low viscosity resin. Semi-thin (1 μm) and ultrathin sections (70 nm) were cut on a Reichert Ultracut S microtome, the latter with a Diatome diamond knife. Semi-thin sections were stained with toluidine blue and examined with a Leica DMR compound microscope, while ultrathin sections were stained with uranyl acetate and lead citrate and examined with either a Philips CM100 or Zeiss EM 902 transmission electron microscope.

### Histology

*Osedax rubiplumus*, *O. frankpressi*, and *O*. “yellow collar” were fixed in 2% paraformaldehyde in 0.1 mol L^−1^ Sorensen’s phosphate buffer with 0.3 mol L^−1^ sucrose at 4°C for 1–2 h, rinsed, and stored in the same buffer as used for fixation until dehydration in a series of graded ethanol dilutions, rinsed with xylene three times for 10 min each, and embedded in paraffin (Paraplast) wax. Serial sections of 7 μm were cut on a rotary microtome and mounted on gelatin-coated glass microscopic slides. Several slides from the ovisac region were de-paraffinized in xylene, rehydrated through a graded series of ethanol, rinsed with 1× phosphate-buffered saline containing 0.2% Triton X-100 (PBS-TX), incubated with Hoechst 33342 (1 μg mL^−1^ in PBS-TX) for 10 min, and rinsed again three times with PBS-TX. Stained sections were mounted in Citifluor AF2 and examined with a Zeiss AxioObserver Z1 epifluorescence microscope or a Leica DMR fluorescent microscope. Micrographs were taken with a Zeiss AxioCam HRm Rev. 3 camera using the Zeiss Axiovision software, or using a Canon Rebel Ti2 SLR camera (Leica DMR).

After being embedded in paraffin, the ovisac region of a specimen of *O. frankpressi* was serially sectioned starting with the posterior of the trunk. Sections were de-paraffinized in three washes of xylene, rehydrated in a graded series of ethanol dilutions, stained with Gill’s hematoxylin (Sigma–Aldrich) and alcoholic eosin Y (Sigma–Aldrich), mounted with DPX (EMS), and cover-slipped. Sections were examined using a Leica DMR compound microscope. For the reconstruction of the ovisac, pictures were taken using the 10× objective and a Canon Rebel T2i. One hundred sixty sections were used for reconstruction with the software program AMIRA® (version 5.4.0). The dorsal and the ventral blood vessels and the oviduct, oocytes, and gonads were outlined manually in each section, and then their surfaces were generated by the software.

## Results

### Female reproductive system

**Figure 1:**
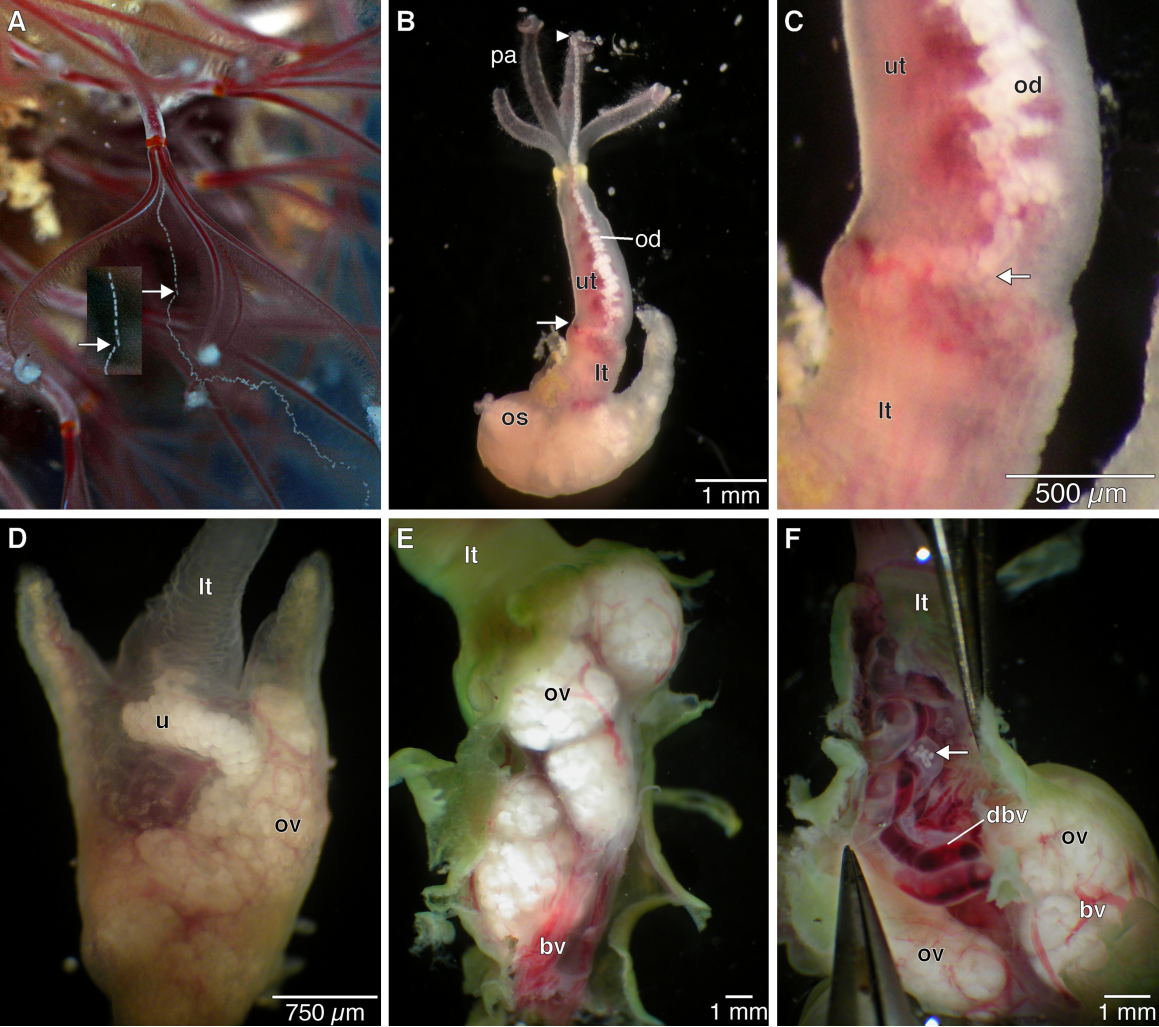
Gross anatomy and morphology of *Osedax* females. A. *Osedax* “orange collar” female spawning *in situ*. The arrow marks opening of the oviduct to the sea. B. *Osedax* “orange collar” female with a highly contracted trunk showing the anterior plume with 4 palps and the end of the oviduct filled with presumably fertilized oocytes (arrowhead); the contracted oviduct running dorsally along the upper trunk is also filled with oocytes. The oviduct moves inside at the beginning of the lower trunk (arrow). C. *Osedax* “orange collar” close up showing the anterior oviduct relocating from exterior to interior (arrow), marking the border between upper and lower trunk. D. Ovisac and lower trunk region of a female of *O*. *roseus* dissected from bone. Ovarian tissue with blood vessels is visible through the skin, as is the uterus region of the oviduct, which contains numerous presumably fertilized oocytes ready to be released to the sea through the anterior oviduct. E. *Osedax frankpressi* lower trunk and ovisac region, with the ovisac epidermis and trophosome removed, showing ovarian tissue with developing oocytes and blood vessels. F. *Osedax frankpressi* lower trunk and anterior ovisac dissected open showing convoluted oviduct with oocytes (arrow) running in close association with the dorsal blood vessel. bv, blood vessel; dbv, dorsal blood vessel; lt, lower trunk; od, oviduct; os, ovisac; ov, ovaries; pa, palps; u, uterus; ut, upper trunk.

**Figure 2:**
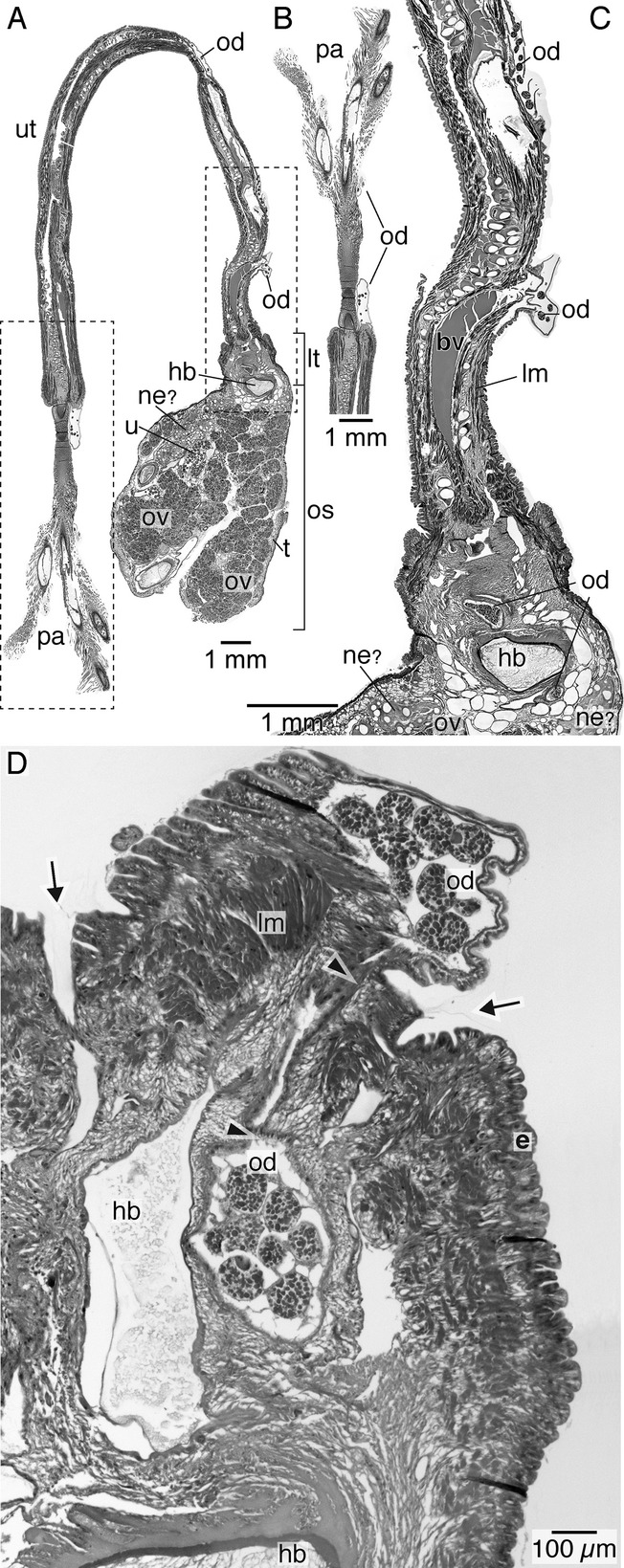
Near sagittal section of *Osedax rubiplumus*. A. Near sagittal section through entire body showing plume, upper trunk, lower trunk, and ovisac region filled with ovaries and developing eggs collected in the uterus. Dashed lines mark areas shown in B and C. B. Detail of crown region showing palps and distal end of the oviduct filled with oocytes. C. Detail of A showing part of the upper trunk with the oviduct running exteriorly and the lower trunk with the oviduct located inside. The thin-walled nonciliated uterus is located further posteriorly. D. A different near sagittal section through the same *O. rubiplumus* female showing the gonopore (arrowheads), where the nonciliated oviduct transitioning from the interior to the exterior (arrowheads), which also marks the border (arrows) of the upper and lower trunk. The anterior part of the heart body in the dorsal vessel in the lower trunk is visible. bv, blood vessel; dbv, dorsal blood vessel; e, epidermis hb, heart body; lm, longitudinal muscle; lt, lower trunk; ne?, putative nephridia; od, oviduct; os, ovisac; ov, ovaries; pa, palps; t, trophosome; u, uterus; ut, upper trunk.

**Figure 3:**
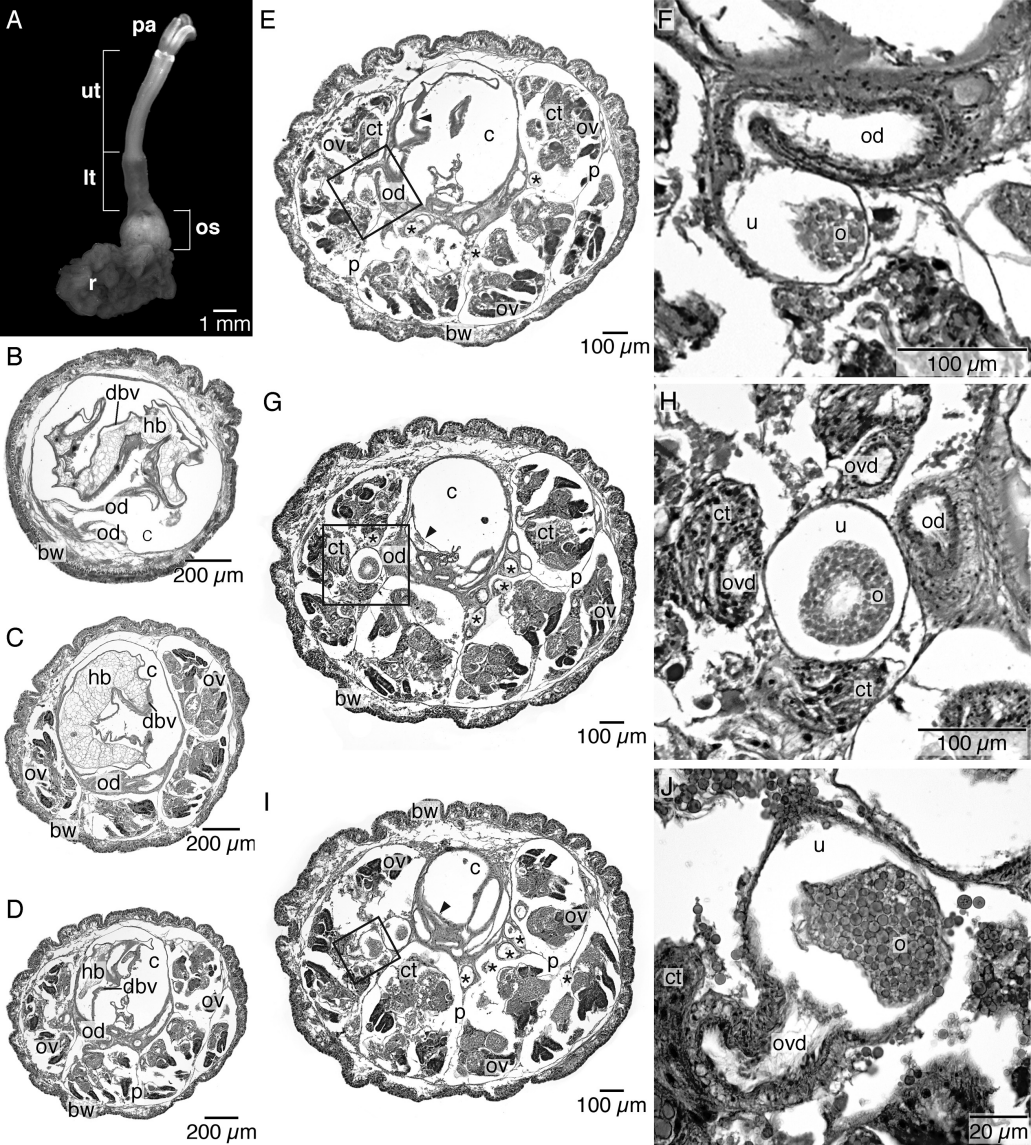
Histology of lower trunk and ovisac region of female *Osedax frankpressi*. A. Actual female specimen of *O. frankpressi* used for 3D reconstruction. B. Cross-section of lower trunk region showing large dorsal blood vessel filled with heart body, and nonciliated anterior oviduct (asterisks). C. Cross-section of anterior ovisac region showing large dorsal blood vessel still filled with heart body, ovarian lobes, and oviduct (asterisks). D. Cross-section of ovisac region more posterior to Fig. 3C, showing dorsal blood vessel (arrowhead) with posterior end of heart body. E. Cross-section of anterior ovisac region showing ovarian lobes separated by peritoneum, ovarian ducts (asterisks), oviduct, and part of base of trunk with dorsal and ventral (arrowhead) blood vessel. The transition between the uterus and anterior oviduct is boxed on the left and shown in F. F. Close-up of part of marked area in Fig. 3E showing the uterus region of the oviduct containing an oocyte, leading into the anterior oviduct. G. Cross-section of ovisac region more posterior than in Fig. 3E, showing several ovarian lobes, with oocytes at various stages of development, several ovarian ducts (asterisks), and the uterus (left) holding a mature oocyte (marked area). H. Close-up of marked area in Fig. 3G, showing the uterus containing an oocyte, connective tissue surrounding an ovarian duct, and cross-sections of the oviduct that comes out from the uterus. I. Cross-section of ovisac region further posterior than Fig. 3E,G, showing several ovarian lobes filling part of the coelom. Several ovarian ducts are indicated (asterisks), as is the transition of the ovarian duct from the ovarian tissue to the uterus (containing an oocyte) in marked area shown in Fig. 3J. J. Close-up of area marked in Fig. 3I showing an ovarian duct from the ovarian tissue connecting to uterus holding an oocyte. arrowhead, dorsal blood vessel; asterisk, ovarian duct; bw, body wall; c, coelom; ct, connective tissue; dbv, dorsal blood vessel; hb, heart body; lt, lower trunk; oc, oocyte; od, oviduct; ovd, ovarian duct; os, ovisac; ov, ovarian lobes; p, peritoneum; pa, palps; r, roots; u, uterus; ut, upper trunk.

We follow the re-orientation of *Osedax* females given in Huusgaard et al. (2012), with a dorso-ventral orientation opposite to previous descriptions. *Osedax* females have a single oviduct that runs from the anterior body to the ovarian tissue in the ovisacs (Fig. 1A–F). The oviduct extends from the extreme dorsal tip of the trunk as a thin, cylindrical, and transparent tube that is unattached to any of the four palps; it opens to the sea anteriorly in the dorsal part of the crown (Figs. 1A,B, 2A,B). In the posterior direction, the thin, transparent oviduct runs along the dorsal surface of the upper trunk (Figs. 1B–D, 2A,C,D). It is hemispherical in cross-section and ciliated along the trunk surface (data not shown). Posteriorly, at a level that we distinguish as the junction between the “upper” and “lower” trunk (Figs. 1B,C, 3A), the oviduct turns inside the trunk (Figs. 1C, 2D) and this point is arguably the gonopore (see below). It expands to become circular, but still runs as a single duct near the dorsal blood vessel (Figs. 1B–D, 2D, 3B,C), although it does appear to show some coiling (Fig. 1B–D). A series of sections of the specimen shown in Fig. 3A show the subsequent expansion of the oviduct into a “uterus” (see below) and then its branching into the actual ovarian tissue (Fig. 3B–J). We describe here the ovarian tissue first.

**Figure 4:**
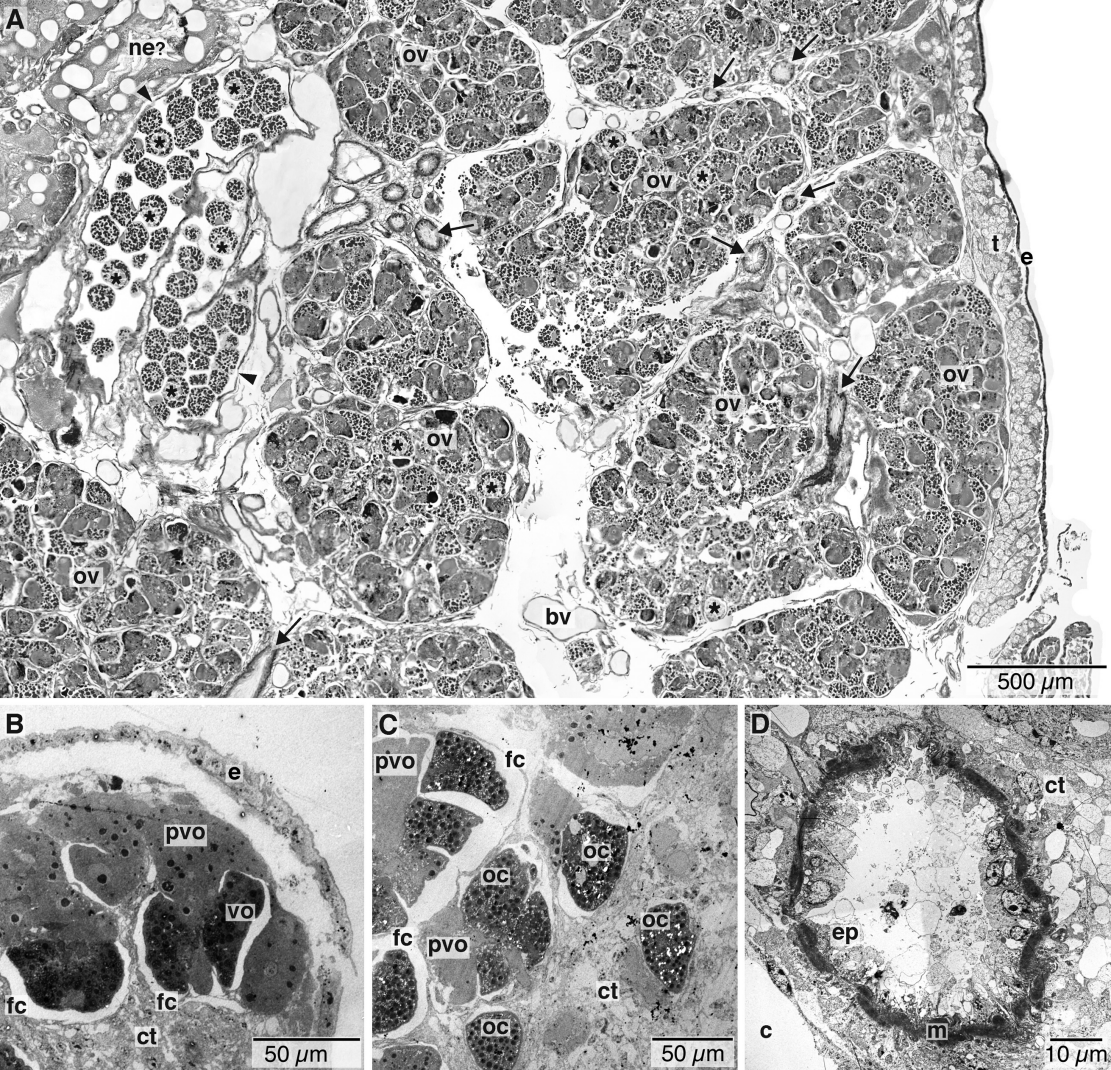
Histology and transmission electron micrographs (TEMs) of ovisac region of *Osedax rubiplumus* and *O*. “green palps.” A. Near sagittal section through the ovisac of *O. rubiplumus* showing ovarian lobes separated by peritoneum. The lobes contain developing oocytes and ensheathing follicle cells. Note the uterus (arrowheads) with mature oocytes (asterisks) and several sections of ovarian ducts (arrows). B. TEM of ovarian tissue of *O*. “green palps” with previtellogenic and vitellogenic oocytes and thin follicle cells with surrounding connective tissue. C. TEM of ovarian tissue of *O*. “green palps” showing oocytes, follicle cells, and connective tissue. D. TEM of ovarian duct of *O*. “green palps” showing single cell-layered epithelium and surrounding musculature. bv, blood vessel; c, coelom; ct connective tissue; e, epidermis, ep, epithelium; fc follicle cells; m, musculature; oc, oocyte; ov ovarian lobes; pvo previtellogenic oocyte; t, trophosome; vo, vitellogenic oocyte.

**Figure 5:**
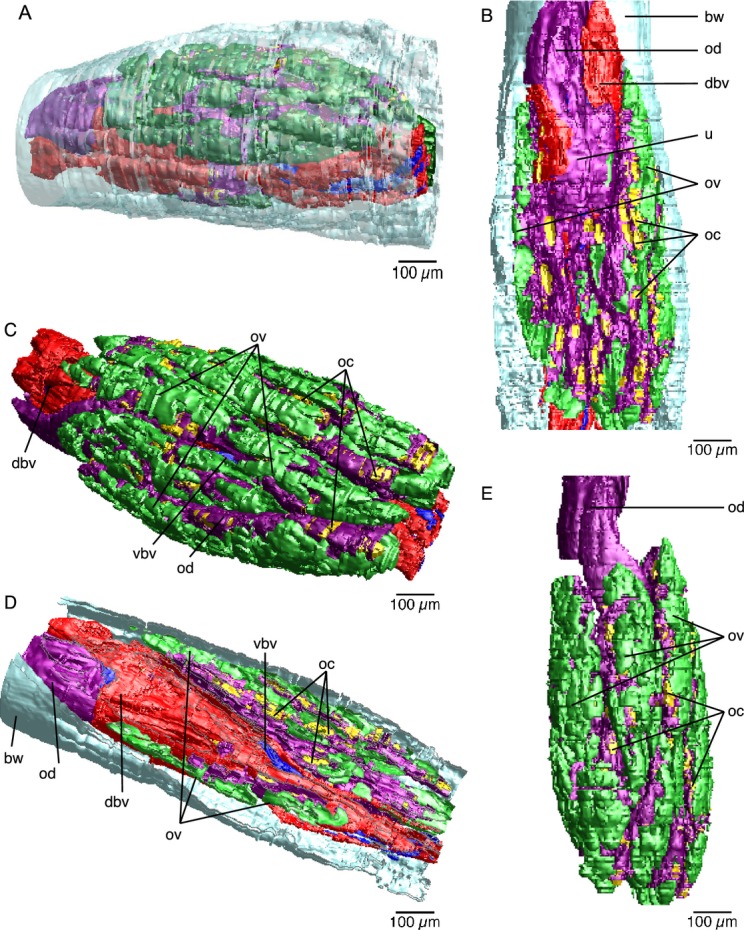
AMIRA® reconstruction of ovisac region of *Osedax frankpressi*. A. 3D model of entire reconstructed ovisac region of *O. frankpressi*. B. Near sagittal section through 3D model of *O. frankpressi* ovisac region, showing oviduct in anterior ovisac that becomes the uterus region posteriorly before branching into ovarian ducts that go into the lobes of ovarian tissue. C. Dorso-lateral view of the ovisac region without the body wall, showing dorsal blood vessel and oviduct running in close association in the center and the ovarian tissue arranged in lobes on the periphery. Oocytes (yellow) are passing from the ovarian tissue to the ovarian ducts. D. Sagittal section through 3D model of *O. frankpressi* ovisac showing large dorsal vessel, oviduct running in close association with it and branching out as ovarian ducts to the ovarian lobes. Late vitellogenic or mature oocytes, scattered through the ovarian tissue, are collected into the ovarian ducts. E. Lateral view of the ovisac region of *O. frankpressi* showing reproductive structures only. Note the oviduct running in the center, ovarian ducts leading to the uterus, and the ovarian tissue arranged in lobes on the periphery. bw, body wall (light blue); dbv, dorsal blood vessel (red); ov, ovarian lobes (green); oc, oocytes (yellow); od, oviduct branching into the ovarian ducts (purple); vbv, ventral blood vessel (dark blue).

The ovarian tissue lies in the coelom of the ovisac region and comprises a large proportion of the female body (Fig. 1D–F). Proliferative germinal epithelia are scattered throughout the ovisac resulting in several ovarian lobes (Figs. 1D–F, 2A). The discrete ovarian lobes were numerous, but could not be counted for this study (Figs. 1D–F, 2A). It is not clear if the ovarian tissue represents multiple ovaries or a single large ovary. The germinal epithelium inside each ovarian lobe grows toward the lumen, forming several stalks (Figs. 3C–E,G,I, 4A,B). Oocytes develop at the distal end of these stalks (Fig. 4A,B). As the oocytes mature, with thin follicle cells surrounding them, they show yolk droplets and increase in size (Fig. 4B,C). Mature oocytes lie at the distal end of the stalks and move into the ovarian ducts, which are present in each lobe (Fig. 3I,J). Each ovarian lobe is surrounded by peritoneal tissue and shows intermingling connective tissue (Fig. 3C–E). The short ovarian ducts are composed of a thick-walled, single cell-layered, nonciliated epithelium surrounded by musculature and embedded in connective tissue (Fig. 4D). The ovarian ducts lead to what we term here as a uterus, an enlarged, thin-walled proximal end portion of the terminal oviduct (Figs. 1D, 2A, 3E–J). The uterus is not surrounded by musculature and lacks cilia. The uterus holds mature oocytes that are likely to be already fertilized (Fig. 1D). From the uterus, the oocytes pass further along to a nonciliated part of the oviduct proper in the lower trunk (Figs. 2A,C,D 3E–J, 4A). As outlined above, the oviduct then emerges at the lower to upper trunk border (at what we interpret as the gonopore, see Discussion) and runs as the exterior oviduct, visible on the dorsal side of the upper trunk (Figs. 1A,B, 2D). The spatial relations of the ovarian lobes, the ovarian ducts, and the oviduct (including the uterus region) with each other, as well as their spatial relation with the dorsal and ventral blood vessels, are shown in a 3-D reconstruction (Fig. 5A–E). Different sagittal views from this reconstruction illustrate the branching system of the reproductive ducts (ovarian ducts, uterus, and oviduct) (Fig. 5B) and the wide dorsal blood vessel and the thinner ventral blood vessel running in close association with each other through the center of the ovisac (Fig. 5D). The female reproductive system only, with the vascular system removed, is shown in Fig. 5E. It resembles a bunch of grapes, with the ovarian lobes arranged on the periphery and the duct system, composed of the ovarian ducts, uterus, and oviduct in the center, corresponding to the stems of the grape cluster.

### Sperm structure

**Figure 6:**
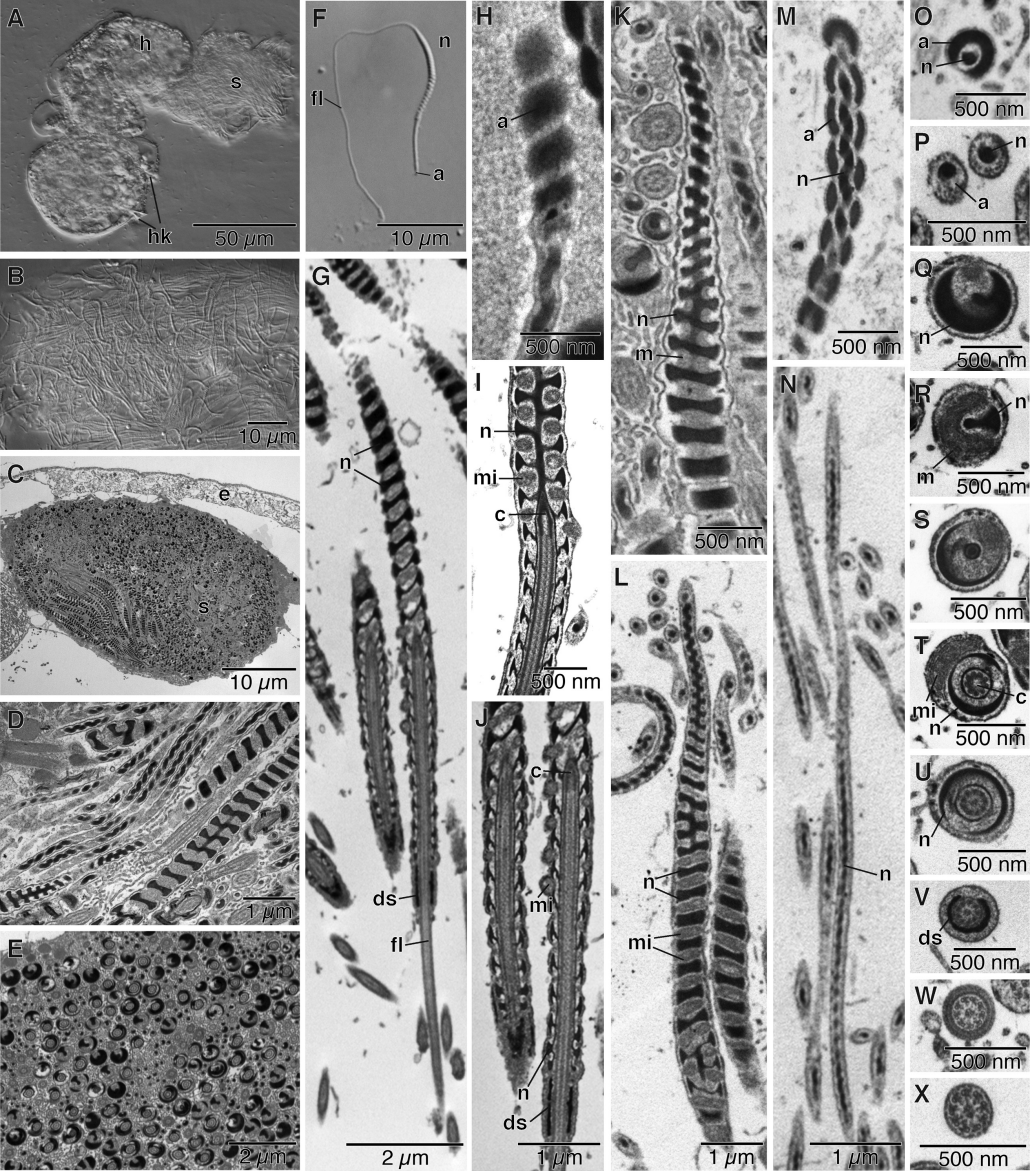
Sperm of *Osedax* “spiral” (A, B) and *O. rubiplumus* (C–X). A. Squeezed *O*. “spiral” male with sperm being ejected from seminal vesicle in the head. B. Unpackaged mature sperm from seminal vesicle of *O*. “spiral.” Note disorganized sperm with spiraled nuclei. C. Transmission electron micrograph (TEM) of a transverse section of the seminal vesicle of an *O. rubiplumus* male showing it filled with free sperm. D. TEM of sperm in seminal vesicle with random orientation and not bundled into spermatophores or spermatozeugmata. E. TEM of seminal vesicle male showing sperm in various cross-sections. F. Interference contrast micrograph of mature sperm showing acrosome region, head comprised of the nucleus and mitochondrial complex, and the tail. G. TEM of a longitudinally sectioned sperm of *O. rubiplumus* showing the base of the head with helically grooved electron-dense nucleus, occupied by mitochondria. The axoneme penetrates into the base of the sperm nucleus and emerges as a free flagellum. Note the small electron dense sheath behind the nucleus. H. TEM of longitudinal section through base of the acrosome vesicle and the thin nucleus. I. TEM of anchoring apparatus for the axoneme, which includes a single centriole. The mitochondrial spiral starts at the level of the anchoring apparatus of this sperm. J. TEM of base of the head of another sperm (from 5G) showing the mitochondrial spiral starts past the anchoring apparatus. Note the small electron dense sheath behind the nucleus. K. TEM of anterior tapering part of nucleus that lacks mitochondria, although still spiral. L. TEM showing the anterior tapering part of nucleus lacking mitochondria transitioning posteriorly to wider helically spiral nucleus occupied by mitochondria. M. TEM of the anteriormost part of nucleus capped by the acrosome. N. TEM of a longitudinal section through longest section seen of tapered, thin anterior nucleus of the sperm head. O. TEM of a cross-section through the acrosome and nucleus. P. TEM of a cross-section through the tapered part of the nucleus, behind the acrosome. Q. TEM of a cross-section through the wider helically spiral nucleus. R. TEM of a cross-section through the wider helically spiral nucleus and an occupying mitochondrion. S. TEM of a cross-section through the nucleus and the anchoring apparatus of the axoneme. T. TEM of a cross-section through the centriole, nucleus, and mitochondrion. U. TEM of a cross-section through axoneme and nucleus. V. TEM of a cross-section through flagellum showing 9×2+2 pattern and the thin electron dense sheath immediately behind the nucleus. W. TEM of a cross-section through the flagellum in the region of the thickened plasma membrane just behind the thin electron dense sheath. X. TEM of a cross-section through free flagellum showing 9×2+2 pattern of the axoneme. a, acrosome; c, centriole; ch, chaetae; ds, dense sheath; e, epidermis; fl, flagellum; h, head; hk, hooks; m, male; mi, mitochondrion; n, nucleus; s, sperm.

In *Osedax* males, the sperm accumulate in a seminal vesicle at the anterior end of the body (Fig. 6A–C). The sperm are densely packed in the seminal vesicle in a seemingly haphazard fashion (Fig. 6C–E). They are clearly not bundled into spermatophores or spermatozeugmata (Fig. 6A–E), but released as free sperm. When a mature male is slightly squeezed, a mass of individual sperm is emitted from the seminal vesicle in the head (Fig. 6A,B,F).

Sperm ultrastructure is described for *O. rubiplumus* and is generally similar in the other *Osedax* species we have observed. Mature sperm (Fig. 6B–X) are filiform, and possess a head region 21 μm in mean length (*n*=9, standard deviation=1.3 μm), and a tail comprised of a flagellum that is up to 32 μm long (Fig. 6F). The head consists of a helical cylindrical nuclear and mitochondrial complex (Fig. 6G–N) that is capped by an apical electron-dense, twisted acrosome (Fig. 6H,M). An anchoring apparatus and a flagellum are embedded basally (Fig. 6G,I,J). The acrosome is a cap-like vesicle that tightly covers the apical part of the nucleus (Fig. 6H,O) for several microns. Although the full length of the acrosome was not measured, it is at least 2.5 μm long (Fig. 6M). No subacrosomal space could be seen. The nucleus immediately proximal to the acrosome is a long uniform cylinder with a helical spiral 0.16 μm thick. The longest section measured was 8.2 μm (Fig. 6M,P), which matches the apparently uncoiled region between the acrosome and thicker part of the sperm head seen in Fig. 6F. The wider (to 0.5 μm in diameter) region of the sperm head is where the mitochondria and nucleus spiral around each other (Fig. 6K,L,Q,R,S). The nucleus shows a deep helical groove that is filled with mitochondrial material, although it was not established if this was a single elongate mitochondrion or a series of mitochondria (Fig. 6I–L,Q,R,S). This nucleus/mitochondrial spiral extends for most of the remaining length of the head. The mitochondrial spiral either terminated at the point of the nucleus where it is penetrated by the flagellum and anchoring apparatus (Fig. 6I), or spiraled around it slightly further (Fig. 6J). The basal part of the nucleus is penetrated by the flagellum and anchoring apparatus for about 4.6 μm, where it forms a thin flange with a raised helical spiral (Fig. 6I,J). The anchoring apparatus is comprised of a single centriole (Fig. 6I,J,T) with an apical cap that is embedded in the base of the nucleus (Fig. 6I,S). At the base of the nucleus, but slightly separated from it, is an electron dense sheath around the axoneme that is less that 1 μm long (Fig. 6G,J,V). The axoneme of the flagellum has a 9x2+2 pattern (Fig. 6U–X).

### Sperm in female *Osedax*

Dwarf males of *Osedax* (Fig. 6A) are normally located in the lumen of the gelatinous tube of the females. The vast majority of males lie in the anterior third of the tube lumen, usually in close vicinity to the oviduct. In the tubes of some females of *O. rubiplumus*, a few males may also be located at the base of the trunk near the ovisac region, a phenomenon also found for males of the species *O*. “spiral,” or even lying outside the ovisac region itself.

**Figure 7:**
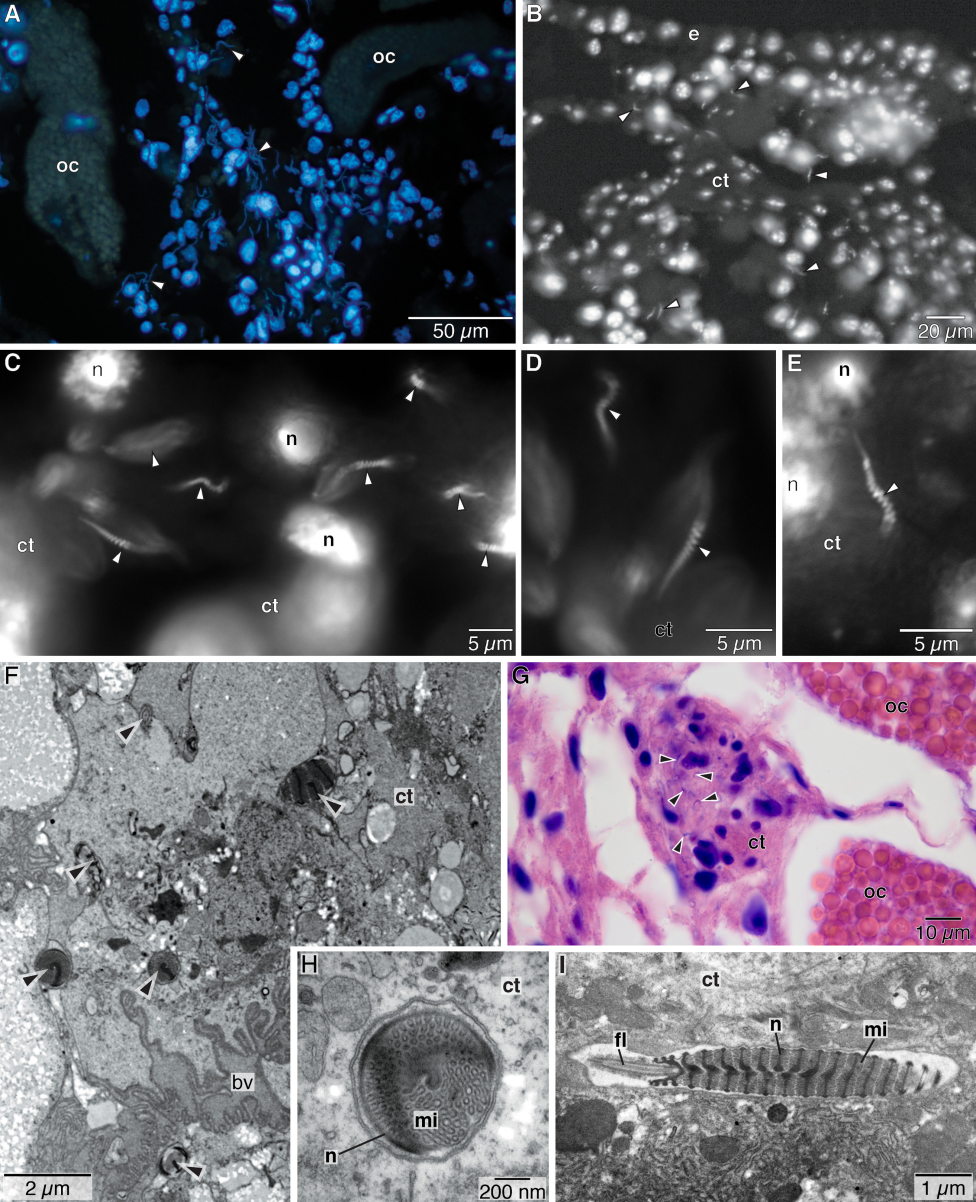
Sperm located in the ovisac region of female *Osedax* spp. A. *Osedax* “yellow collar” cross-section stained with Hoechst 33342 nuclear stain, showing large roundish nuclei of female tissue in blue, autofluorescent yolk of oocytes in pale yellow-green, and thin long sperm (arrowheads) in blue. B. *Osedax* “yellow collar” cross-section more anterior than in 5A stained with Hoechst 33342 nuclear stain, showing large roundish nuclei of female tissue, autofluorescent connective tissue, and thin long sperm (arrowheads). C–E. Sperm (arrowheads) in female *O*. “yellow collar” stained with Hoechst 33342 nuclear stain, showing characteristic helical nucleus of sperm. F–G. 7 μm-cross-section of part of *O*. *frankpressi* ovisac region stained with hematoxylin and eosin, showing sperm (arrowheads) embedded in female connective tissue in ovarian lobe, nuclei in blue. H. Transmission electron micrographs of sperm (arrowheads) in connective tissue of ovisac region of *O. rubiplumus*. I. Cross-section through sperm in connective tissue of *O*. *rubiplumus* showing nucleus and mitochondria. J. Cross-section through connective tissue of *O. rubiplumus* showing part of sagittal sectioned sperm with characteristic helical nucleus and mitochondria. K. Cross-section through connective tissue of *O. rubiplumus* showing part of sagittal sectioned sperm with characteristic helical nucleus, mitochondria, anchoring apparatus, and part of the flagellum. arrowheads, sperm; ct, connective tissue; bv, blood vessel; e, epidermis; fl, flagellum; mi, mitochondrion; n, nucleus; oc, oocyte; ov, ovarian tissue.

Observations of the interior of the oviduct along the trunk revealed no sperm. Similarly, no sperm were found stored in the uterus (Figs. 1D, 5). However, Hoechst nuclear staining on paraffin sections and TEM revealed many sperm throughout the ovisac of the investigated females (Fig. 7A–E). The sperm were associated with tissue near the ovarian ducts (Fig. 7A–G). We were able to document the presence of sperm in the ovisac of females of *O. rubiplumus*, *O*. *frankpressi*, *O*. “green palps,” and *O*. “yellow collar.”

While in some instances sperm tended to be located among the cells of the connective tissue, in other instances, the sperm seemed to be located in invaginations of such cells (Fig. 7H–I). In all cases, the sperm were always found in close vicinity to the oocytes (Fig. 7A,F). Several spermatozoa occurred in close vicinity to each other and several such groups were found throughout the ovisac region of the females (Fig. 7A,B,G). The sperm within such clusters were not oriented in the same way, but faced in different directions (Fig. 7C).

### Other anatomical observations

With reference to the circulatory system, major blood vessels were found in the anterior portion of the ovisac (Figs. 1F, 5). These branch out to form a ramifying network that surrounds and extends into the ovarian tissue as well as down into the root system (Fig. 1D–F). A tissue mass that we interpret to be the heart body (sometimes referred to as the corpus cardiacum) was found lying in the dorsal blood vessel in the posterior part of the lower trunk and the anterior part of the ovisac (Figs. 2, 3B–D). Tissue that lies at the base of the lower trunk and extends posteriorly into the ovisac, but outside the ovarian tissue, is of unknown function. It is possible that this could be nephridial (Figs. 2A,C, 4A). Owing to the contracted nature of the specimens sectioned, we were not able to trace any ducts to the exterior, although the putative nephridial tissue does appear to be associated with blood vessels (Figs. 2A,C, 4A).

## Discussion

We examined the ovisac region of several females of four different *Osedax* species (*O. rubiplumus*, *O. frankpressi*, *O*. “green-palps” and *O*. “yellow-collar”) and all showed the same pattern. Sperm structure was similar in all cases and males had free sperm lying in their seminal vesicles. We also documented the presence of sperm in the ovarian tissue of these four taxa.

### Female reproductive system

The female reproductive system in *Osedax* seemingly differs from the organization in other siboglinids, but close examination suggests that there are possible homologies (Table 1). *Osedax* females possess one unpaired ciliated oviduct opening in the crown region. The oviduct runs along the outside of the dorsal side of the trunk. At the junction of the upper and lower trunk, the oviduct moves inside, becomes nonciliated, and then widens to become a “uterus,” where the fertilized oocytes are held until they are spawned. The term ovisac has been consistently used for *Osedax* to refer to the whole body region containing the gonads (Rouse et al. 2004, 2008; Glover et al. 2005; Fujikura et al. 2006), so we here use the term “uterus” for this oviduct region storing the fertilized oocytes (Fig. 1D). This uterus is arguably homologous with the ovisacs in Vestimentifera (Webb 1977; Hilário et al. 2005), and similar areas seem to be present in Frenulata and *Sclerolinum* (Ivanov 1961; Eichinger et al. 2013). We suggest that, for females of *Osedax*, the point where the oviduct exits from the lower trunk (Fig. 1B,C) is positionally the same as the gonopores found in other siboglinids. We therefore call this exit point the gonopore, rather than using this term for the opening at the extreme tip of the oviduct at the level of the crown of palps (Fig. 1A,B). In Vestimentifera and *Sclerolinum*, the gonoducts exit at the anterior end of the trunk, near the junction of the forepart or vestimentum (Ivanov 1963; Webb 1969; Eichinger et al. 2013), except for female frenulates where they open more posteriorly on the trunk (Ivanov 1963). Two grooves in the vestimentum carry the male gametes forward in male Vestimentifera. These grooves are not present in the females (Webb 1977; Jones 1988). However, in both sexes, the vestimental folds act as a tube that helps to get the gametes to the outside water from the tube (Webb 1977), and there are similar folds in *Sclerolinum* (Eichinger et al. 2013).

If the emergence of the oviduct in *Osedax* females on the lower trunk is positionally the same as the gonopore in other siboglinids, then the thin-walled oviduct found along the trunk (Fig. 1A,B) can be interpreted as a covered groove extending from the gonopore. The external oviduct of *Osedax* may therefore be homologous to the grooves found in vestimentiferan males that carry gametes forward from the tube. Making sure that fertilized oocytes are expelled from the tube is critically important for *Osedax* females. If retention of fertilized oocytes occurs, then dwarf males might ensue from their own retained larvae with consequent inbreeding, as environmental sex determination is a likely feature of their reproductive mode (Rouse et al. 2008; Vrijenhoek et al. 2009). Avoiding this scenario would explain why the oviduct in most *Osedax* species extends anteriorly as part of the crown of palps (Fig. 1A,B), a feature not seen in any other siboglinids. The exception among described *Osedax* to date is *O. japonicus* Fujikura,Fujiwara & Kawato 2006, which has only a short oviduct projecting beyond the trunk (Fujikura et al. 2006). This species has been proposed to brood embryos (Fujikura et al. 2006; Miyamoto et al. 2013).

As with the ovisac in other siboglinids, the uterus of *Osedax* is thin-walled and nonciliated. The uterus then branches out as ovarian ducts in the ovarian lobes to collect mature oocytes. Owing to the convoluted nature of the female gonad, we were unable to determine if *Osedax* has a single ovary, as in *Sclerolinum* (Eichinger et al. 2013), or if it has paired structures as seen in most Vestimentifera and Frenulata (Table 1). All other siboglinids have the oviduct entering the ovary at the posterior end, thus making the female reproductive system U-shaped (Ivanov 1961; Webb 1977; Eichinger et al. 2013), while in *Osedax*, the uterus was found opening near the anterior end of the ovarian tissue. Further resolution of the nature of the female gonad of *Osedax* will require the study of early developmental stages of females. We found that oogenesis in *Osedax* appears to be similar to that seen in other siboglinids, in that it is intraovarian, with associated follicle cells. The only temporal observations on spawning by *Osedax* to date found that females released an average of 335 eggs per day, but that the number of oocytes spawned per day varied greatly, suggesting that not all the females spawned daily (Rouse et al. 2009). Nevertheless, this does suggest that *Osedax* undergoes continuous oogenesis and the various stages of oogenesis we observed in all specimens sectioned support this.

### Sperm structure

The sperm of *O. rubiplumus*, *O. frankpressi*, *O*. “spiral,” and *O*. “yellow-collar” were all similar and shared features with those of other siboglinids. The sperm are elongate with an apical coiled acrosome capping the nucleus, which is a very narrow spiral of marked electron density. The distal part of the nucleus is also electron dense and has a deep helically spiraled groove occupied by mitochondria, resulting in there being no midpiece, and the tail is simply a flagellum. Where ultrastructural studies have been performed on siboglinids, the mature sperm are markedly similar to this description (Franzén 1973; Gardiner & Jones 1985; Southward & Coates 1989; Southward 1993; Marotta et al. 2005; Eichinger et al. 2013). However, the spiral of the mitochondria in *Osedax*, *Sclerolinum*, and Vestimentifera is marked by being embedded in a deep helical groove of the nucleus, a feature not seen in Franzén (1973) description of the sperm of *Siboglinum*, where the mitochondria simply wrap around the slightly spiraled nucleus. *Osedax* sperm also differ from those of Vestimentifera in not having an apical region of the nucleus with less electron density.

*Osedax* sperm appear to be unique among siboglinids in that the sperm axoneme penetrates the base of the nucleus for 4–5 μm. The mature sperm of *Osedax* males are collected in the anterior seminal vesicle, but are not bundled into spermatophores as in frenulates, or aggregated as spermatozeugmata as in Vestimentifera (Ivanov 1963; Southward 1993; Marotta et al. 2005). In vestimentiferan and frenulate males, the spermatozeugmata or spermatophores are stored in small seminal vesicles just behind the gonopore (van der Land & Nørrevang 1977; Webb 1977). Little is known on the reproductive biology of *Sclerolinum*, but their sperm are also not bundled into spermatophores (Southward 1993; Eichinger et al. 2013), and they may be released to the water, or otherwise transferred to the female, as free sperm or as bundles (Southward et al. 2005) that may be spermatozeugmata.

### Sperm storage by females and fertilization

The spermatozeugmata or spermatophores of Vestimentifera and Frenulata reach the females either by direct contact or passive transport through the water column and stick to the outside of the females in vicinity of the gonopore. The presence of sperm in the female genital tract of Vestimentifera was reported by Gardiner & Jones (1985), who suggested that fertilization was internal; this has been further documented by Hilário et al. (2005), Drozdov & Galkin (2012), and Karaseva et al. (2012). Malakhov et al. (1996) described ciliated “funnels” connecting the posterior oviduct to the ovary and observed those funnels to be filled with sperm. It seems most likely that these “funnels” are the same structure described as outpocketings of the very posterior end of the oviducts (near the ovary) and termed spermathecae by Hilário et al. (2005) and Karaseva et al. (2012). Sperm storage has not been documented in Frenulata, although internal fertilization is at least known for *Siboglinum fiordicum* (Bakke 1976), and is suggested to be general (Southward 1993). Nothing is known about fertilization or larval development in *Sclerolinum*, although the similarity of its sperm structure to that of other siboglinids (Eichinger et al. 2013) suggests that fertilization may be internal, but how the sperm get to the female remains to be resolved.

None of the *Osedax* females investigated here showed evidence of sperm storage along the main oviduct or inside the uterus. Indirect evidence for internal fertilization in *Osedax* had previously been provided from the fact that females released fertilized eggs, which started developing a few hours after release (Rouse et al. 2008). The discovery of sperm throughout the ovarian tissue of female *Osedax* clearly corroborates the earlier hypothesis of internal fertilization (Rouse et al. 2008, 2009). The use of the fluorescent Hoechst nuclear stain on paraffin sections (Fig. 7A–E) allowed us to identify the sperm based on the characteristic helical nucleus. Using a fluorescent stain was valuable, as the sperm were not located in large clusters or along the lumen or margin of the oviduct. Being small, unpackaged, but located within tissue near the ovarian ducts of the female makes it hard to see individual sperm, even in hematoxylin and eosin-stained sections (Fig. 7G), which usually provide good contrast between nuclei and other tissues. In contrast with Vestimentifera (Hilário et al. 2005), sperm are found in the ovarian tissue of female *Osedax*, rather than the oviduct. Also, the sperm are associated with, or incorporated within, the connective tissue near the ovarian ducts and are not in the lumen of the oviduct. Sperm may be found throughout the whole ovisac area. Our observation of sperm in the connective tissue suggests that fertilization most likely takes place in the ovarian ducts, before the oocytes are moved to the uterus. Positionally, this fertilization site is only slightly further back than the sperm storage area known for Vestimentifera (Hilário et al. 2005).

It is still unclear how sperm enter *Osedax* females and then end up in the ovary. Males of *Osedax* are mainly found lying in the tube lumen, near the oviduct along the anterior trunk region of females (Rouse et al. 2008; Vrijenhoek et al. 2009). For *O*. *rubiplumus*, the maximum reported harem size of males living with a female was 607 males, with an average of around 27 for spawning females (Vrijenhoek et al. 2008). Even though individual males were located near the ovisac region in some material examined for this study, the majority of males were found in the anterior part of the female tube. We propose that the sperm of the males lying near the oviduct region of the trunk are able to penetrate the thin oviduct. The seminal vesicle of the male head has an exit dorsally that may also serve as an intromittent organ (Rouse et al. unpubl. data). Once inside the oviduct, the sperm may swim down the oviduct to the ovarian tissue where they are then stored. Sperm storage within cell invaginations is also found in some members of the polychaete family Sabellidae. However, within these groups, the sperm are usually stored in epidermal cells (Rouse 1996). Tissue penetration by individual sperm for fertilization is also found in the salp *Thalia democratica* (Forskål 1775), which also possesses elongate sperm with a mitochondrion wrapped around a helically spiraled nucleus (Holland 1988; Boldrin et al. 2009).

Direct transmission of sperm from the male to the female eliminates the need for packaging the sperm in spermatozeugmata or spermatophores for transfer. The occasional males found around the ovisac region face a problem in transmitting their sperm, in that it would have to penetrate through the epidermis and trophosome to reach the ovary. It may be that these males were simply misplaced, or transported down from the anterior part of the tube away from the oviduct. However, it should be noted that hypodermic sperm transfer is seen across a range of annelids, e.g., in *Trilobodrilus axi* Westheide 1967 (Dinophilidae), *Myzostoma cirriferum* Leuckart 1836 (Myzostomida), and Hirudinea (Ax 1968; Eeckhaut & Jangoux 1991; Rouse 1999; Sella 2006; Świątek et al. 2007), and may also occur in *Osedax*. Of relevance in this regard is the observation that sperm heads were able to penetrate the cuticle and epidermis of the trunk of the frenulate *Siboglinum fiordicum* (Flügel in Southward 1999).

### Heart body and putative nephridia

A heart body (intravasal body or corpus cardiacum) is known to occur in the dorsal blood vessel of many different annelid groups such as cirratuliforms, terebelliforms, sabellariids, arenicolids, some serpulids, and also in most siboglinids (Rouse & Fauchald 1997; Rouse & Pleijel 2001). The heart body generally lies anteriorly and can extend along the dorsal blood vessel for several segments. Functions proposed for this organ include blood production (hemopoiesis), acting as a valve controlling the direction of blood flow in the dorsal vessel, and the accumulation of waste (Kennedy & Dales 1958; Braunbeck & Dales 1985). Where the appropriate histological studies have been made, a heart body has been documented in the dorsal vessel of some frenulates, *Sclerolinum*, and most Vestimentifera (except *Tevnia jerichona* Jones 1985) (Ivanov 1963; Jones 1981; Southward 1993; Schulze 2002; Eichinger et al. 2013). Functions such as acting as a valve and hemoglobin metabolism have been suggested for the siboglinid heart body (Jones 1988; Schulze 2002). In Vestimentifera, the heart body starts in the anterior vestimentum and continues along the trunk and into the opisthosoma (Schulze 2002). In some species, the heart body can be prominent and may occlude the lumen of the blood vessel; in others, it is small and relative constant in size. Once in the opisthosoma, the vestimentiferan heart body becomes a very narrow strand of tissue that is only visible via transmission electron microscopy (Schulze 2002). In frenulates, the heart body is present in the trunk only (Ivanov 1963) and in *Sclerolinum*, the heart body also extends along the entire length of the trunk to the opisthosoma (Eichinger et al. 2013). Our results show that *Osedax* females have a heart body in the dorsal vessel at the base of the lower trunk and in the anterior part of the ovisac, but observations by light microscopy did not reveal it more posteriorly within the ovisac. The spongy appearance of the heart body is similar to that seen in other siboglinids (Ivanov 1963; Schulze 2002). The location of the heart body further corroborates the designation of the dorsal vessel as in Huusgaard et al. (2012).

Rouse (2001) interpreted the trunk region of siboglinids as representing either the bulk of the first segment, or as a separate elongate second segment, with the vestimentum, or frenulum region, representing part or all of segment 1. The former view is supported by the recent review by Bright et al. (2013). If the position of the heart body and the emergence of the oviduct onto the trunk can provide a guide to the regionalization of *Osedax*, then this would suggest that the majority of the trunk is equivalent to the vestimentum/forepart region of the other siboglinids. This was previously proposed by Rouse et al. (2008), based on the structure of the nervous system of female *O. roseus* Rouse,Worsaae,Johnson,Jones & Vrijenhoek 2008.

In frenulates, a pair of nephridia, each with its own dorsal excretory pore, lie near the anterior end between the palp bases and the frenulum (Ivanov 1963; Southward 1993). In Vestimentifera, the pair of nephridia also open dorsally, via either one or two pores, at the anterior margin of the vestimentum (Schulze 2002; Karaseva et al. 2012), with the main part of the nephridial organs lying posterior to the brain (Gardiner & Jones 1993). Recent morphological studies on *Sclerolinum contortum* Smirnov 2000 could not identify excretory structures (Eichinger et al. 2013). Our identification of putative nephridia in the anterior ovisac region is somewhat at odds with our interpretation of the trunk of *Osedax* as equivalent to the vestimentum/forepart region of other siboglinids. In these groups, the nephridia occur in the vestimentum/forepart, which would suggest that the nephridia should be found in the anterior trunk of *Osedax*, although we found no trace of them in this region. Further study of well-fixed relaxed specimens of *Osedax* is required to resolve this issue.
